# The first data on the freshwater microcrustaceans of Shokalsky Island (Russian Arctic)

**DOI:** 10.3897/BDJ.4.e10930

**Published:** 2016-12-23

**Authors:** Anna Novichkova

**Affiliations:** 1 Lomonosov Moscow State University, Moscow, Russia; 2 A.N. Severtsov Institute of Ecology and Evolution, Moscow, Russia

**Keywords:** Cladocera, Copepoda, microcrustaceans, freshwater, Russian Arctic, Shokalsky Island

## Abstract

**Background:**

Information on freshwater invertebrates of the Russian Arctic is very scarce, especially concerning insular biota. The species composition of microcrustaceans (Cladocera, Copepoda) of many arctic islands is still unknown and have never been explored. Here we report the results of the first investigation of the zooplankton of the Shokalsky Island (Yamalo­Nenets Autonomous Okrug, Russia).

Information on freshwater invertebrates of the Russian Arctic is very scarce, especially concerning insular biota. The species composition of microcrustaceans (Cladocera, Copepoda) of many arctic islands is still unknown and have never been explored. Here we report the results of the first investigation of the zooplankton of the Shokalsky Island (Yamalo­Nenets Autonomous Okrug, Russia).

**New information:**

The new records reported here are novel for the region and significantly expand the knowledge of the high­-latitude aquatic biota. We studied the species composition of Cladocera and Copepoda of 21 freshwater habitats located on the south­western part of Shokalsky Island. We found 15 species of microcrustaceans in total and all of them are reported for the first time here. Also, the obtained data expand the existing ranges of distribution of some species and report several new taxa for the whole Yamalo­Nenets region of Russia.

## Introduction

**Figure 1.**
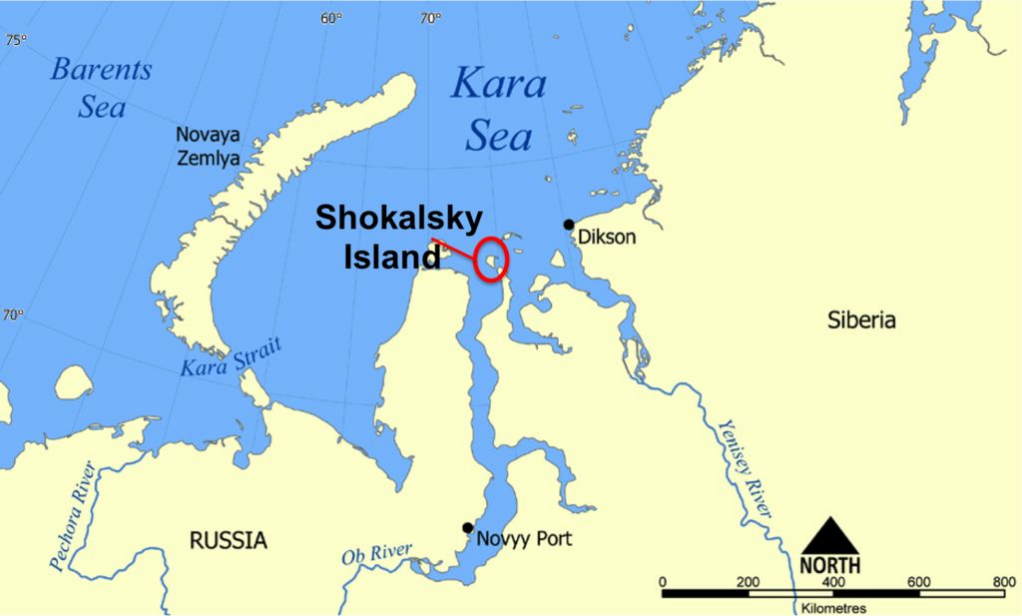
Location of the Shokalsky Island on the map. Original map from: commons.wikimedia.org.

Shokalsky Island is a small island in the Kara Sea located in the Yamalo­Nenets Autonomous Okrug of Russia (Fig. 1). It is separated from the mainland by a narrow strait, which is only 5­9 metres in width. The island is a flat plain covered by tundra with a great number of rivers, small lakes and ponds (Il'yna et al. 1985). It belongs to the Gydan Nature Reserve, which includes a diverse terrain of northern Gydan Peninsula, preserving waterfowl nesting areas, polar bear, and walrus and is known as the nothermost nature reserve in Western Siberia (Nikiforov 1997). Due to its remotness and very limited access to some territories, the level of scientifical researches here is rather low and some of the aspects are under studied. Despite the long period of freshwater observations in the region, invertebrate fauna of all the peninsulas lying in the Yamalo­Nenets Autonomous Okrug is known only fragmentarily (Sharapova and Abdullina 2004). According to the latest data, information on the zooplankton of the northen Yamal is very scarce, while the species inhabiting water bodies of Tazovsky and Gydan Peninsulas are known only from rare publications focused on large lakes and rivers (Popov 2012). The species composition of the microcrustaceans (Cladocera, Copepoda) of Shokalsky island has never been explored.

## Materials and methods

**Figure 2.**
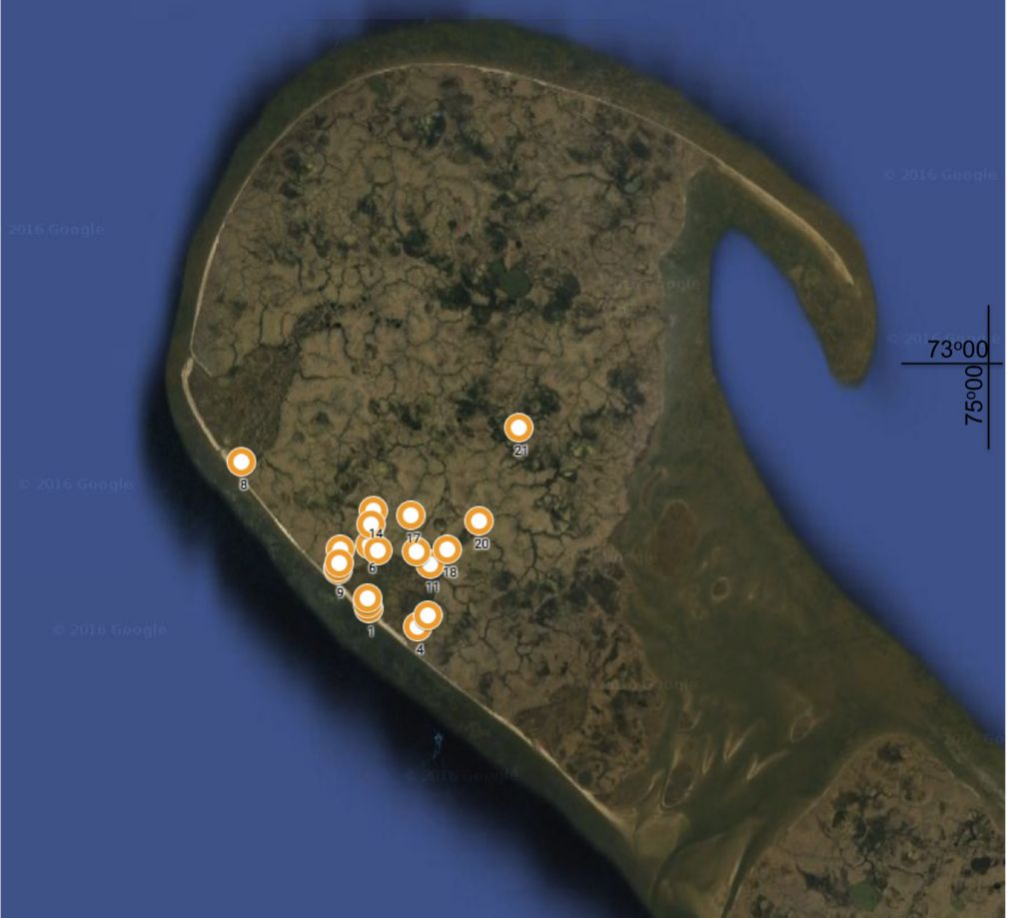
Location of the sampled stations in inner water bodies on the map of Shokalsky Island *(Google Maps)*.

The samples were collected during a hydrobiological survey of the compex expedition of KUBZ (Moscow Zoo young biologist's coterie) in August, 2014. Microcrustaceans were collected from 21 freshwater habitats from south­western part of Shokalsky Island, most of them were small thermokarst ponds with the depth of 0.5­ - 1.5 m (Fig. 2). Environmental variables such as bottom sediment type (clay, silt, sand, detritus or thick mosses, measured in accordance with Wentworth Grade Scale (Ongley 1996)), average depth (mean value for the whole sampled area) and size (average length of diameters) of the water body were noted for each site (Table 1). The sampling was performed from the shore using a qualitative plankton net (type “Apstein”, mesh size 50 μm). Upon collection, all samples were preserved in ethanol (96%). Species identification and enumeration was carried out primarily in Bogorov counting chambers; the total numbers of Cladocera and Copepoda were recorded. Description of the distributional ranges of the species is also provided in the checklist: AT - Afrotropical, AU - Australasian, ANT - Antarctic, NA - Nearctic, NT - Neotropical, OL - Oriental, PA - Palaearctic, PAC - Pacific oceanic islands.

## Checklists

### List of species Cladocera and Copepoda recorded on Shokalsky Island

#### Cladocera

Latreille, 1829

#### Chydoridae

Dybowski et Grochowski, 1894

#### Acroperus harpae

(Baird, 1834)

##### Notes

localities no. 1, 7. **Distribution**: AT, AU, NA, NT, OL, PA.

#### Alona affinis

(O.F. Müller, 1776)

##### Notes

localities no. 1, 3, 5, 6, 7, 16, 21. **Distribution**: AT, AU, NA, NT, OL, PA.

#### Alona quadrangularis

(Leydig, 1860)

##### Notes

locality no. 9. **Distribution**: AT, AU, NA, OL, PA.

#### Chydorus sphaericus

(O.F. Müller, 1776)

##### Notes

localities no. 1-9, 11, 13, 16, 19, 20, 21. **Distribution**: AT, AU, NA, NT, OL, PAC, PA.

#### Graptoleberis testudinaria

(Fischer, 1848)

##### Notes

locality no. 17. **Distribution** (subsp. *testudinaria*): AT, AU, NA, NT, OL, PA.

#### Eurycercidae

Kurz, 1875 sensu Dumont et Silva-Briano, 1998

#### Eurycercus (Teretifrons) glacialis

Lilljeborg 1887

##### Notes

localities no. 1, 21. **Distribution**: NA, PA.

#### Daphniidae

Straus, 1820

#### Daphnia cf. pulex

Leydig, 1860

##### Notes

localities no. 3, 5, 6, 7, 13, 19. **Distribution**: AT, NA, NT, PA.

#### Scapholeberis mucronata

(O.F. Müller, 1776)

##### Notes

localities no. 1, 6, 7. **Distribution**: NA, NT, PA.

#### Simocephalus vetulus

(O.F. Müller, 1776)

##### Notes

locality no. 1. **Distribution**: PA.

#### Bosminidae

Baird 1845 sensu Sars 1865

#### Bosmina (Bosmina) longirostris

(O. F. Müller, 1785)

##### Notes

localities no. 17, 18. **Distribution**: : AT, ANT, AU, NA, NT, OL, PAC, PA.

#### Sididae

Baird, 1850

#### Latona setifera

(O.F. Müller, 1776)

##### Notes

localities no. 21. **Distribution**: NA, PA.

#### Polyphemidae

Baird, 1845

#### Polyphemus pediculus

(Linnaeus, 1761)

##### Notes

localities no. 1-9, 13, 14, 20, 21. **Distribution**: NA, PA

#### Copepoda

Milne Edwards, 1840

#### Cyclopoida

Burmeister, 1834

#### Cyclopidae

Rafinesque, 1815

#### Cyclops vicinus

Uljanin, 1875

##### Notes

localities no. 1, 4, 18, 19.

#### Calanoida

Sars G.O., 1903

#### Diaptomidae

Baird, 1850

#### Diaptomus cf. castor

(Jurine, 1820)

##### Notes

locality no. 1. **Distribution**: PA (Europe (Austria, France....), Greenland, Northern Alaska (Colville River)).

#### Leptodiaptomus angustilobius

(Sars G.O., 1898)

##### Notes

localities no. 1-9, 12, 13, 14, 18, 20, 21. **Distribution**: NA (Arctic and Subarctic Canada, to the Kuril Islands).

## Discussion

In total 15 species of microcrustaceans were identified, comprising 12 species in 12 genera of Cladocera, and three species in three genera of Copepoda. All of the taxa have not been previously documented on the island. Microcrustaceans were found in 90% of the studied sites. The number of species encountered in each water body varied from one to ten (Table 2). The most common species in the studied sites were *Leptodiaptomus angustilobius* (Sars, 1898), *Polyphemus pediculus* (Linnaeus, 1761) and Chydorus cf. sphaericus (Muller, 1776), they usually dominate in the communities and occured in most of the investigated water bodies.

The distributional ranges of all the species are rather wide, none of them are restricted to the arctic area or more limited region. The areas of the species are noted in the Checklist according to the FADA Databases of Cladocera (Kotov et al. 2013) and Copepoda (Boxshall and Defaye 2009). The most important findings are *Latona setifera* (Muller, 1776), Diaptomus cf. castor (Jurine, 1820) and *Graptoleberis testudinaria* (Fischer, 1848). The first two species have never been found on the territory of Yamalo-Nenets Autonomous Okrug, and the third one was only known from waters of lower Ob' River (Semyonova et al. 2000). All of them occured rarely in separate water bodies. For the species *L. setifera* this record is the northernmost finding ever (Korovchinsky 2004).

## Supplementary Material

XML Treatment for Cladocera

XML Treatment for Chydoridae

XML Treatment for Acroperus harpae

XML Treatment for Alona affinis

XML Treatment for Alona quadrangularis

XML Treatment for Chydorus sphaericus

XML Treatment for Graptoleberis testudinaria

XML Treatment for Eurycercidae

XML Treatment for Eurycercus (Teretifrons) glacialis

XML Treatment for Daphniidae

XML Treatment for Daphnia cf. pulex

XML Treatment for Scapholeberis mucronata

XML Treatment for Simocephalus vetulus

XML Treatment for Bosminidae

XML Treatment for Bosmina (Bosmina) longirostris

XML Treatment for Sididae

XML Treatment for Latona setifera

XML Treatment for Polyphemidae

XML Treatment for Polyphemus pediculus

XML Treatment for Copepoda

XML Treatment for Cyclopoida

XML Treatment for Cyclopidae

XML Treatment for Cyclops vicinus

XML Treatment for Calanoida

XML Treatment for Diaptomidae

XML Treatment for Diaptomus cf. castor

XML Treatment for Leptodiaptomus angustilobius

## Figures and Tables

**Table 1.** Locations and dates of sampling with notes on the water body type and substratum (bottom sediment).

**Site No.**	**Date**	**Coordinates**	**Altitude**	**Water body type**	**Bottom Sediment**	**Average Size**	**Average Depth**
1	03.08.2014	72.91667°N, 74.33418°E	7m	bayou pond	silt, mosses	5m x 13m	0.5m
2	03.08.2014	72.91928°N, 74.33338°E	2m	bayou pond	silt	5m x 10m	0.5m
3	03.08.2014	72.92120°N, 74.33328°E	-6m	bayou pond	silt	8m x 20m	0.3m
4	04.08.2014	72.91103°N, 74.39402°E	-1m	thermokarst pond	silt, detritus	6m x 10m	0.5m
5	04.08.2014	72.91523°N, 74.40652°E	7m	thermokarst pond	clay, sand	15m x 30m	1.5m
6	05.08.2014	72.94028°N, 74.33675°E	3m	thermokarst pond	mosses	1.5m x 15m	1.5m
7	05.08.2014	72.94101°N, 74.33377°E		boggy stream	mosses	1m x 1.5m	1.5m
8	06.08.2014	72.96975°N, 74.33328°E	-1m	thermokarst pond	clay, silt	5m x 10m	0.5m
9	07.08.2014	72.93123°N, 74.29818°E	3m	bayou pond	sand, mosses	3m x 5m	0.5m
10	07.08.2014	72.93850°N, 74.30105°E	0m	thermokarst pond	sand, mosses	2m x 5m	1.5m
11	10.08.2014	72.93318°N, 74.40840°E	-1m	thermokarst pond	silt, mosses	5m x 10m	1m
12	10.08.2014	72.93198°N, 74.29845°E	8m	thermokarst pond	sand, mosses	1.5m x 6m	0.5m
13	11.08.2014	72.93358°N, 74.29932°E	3m	thermokarst pond	mosses	2m x 2.5m	1m
14	12.08.2014	72.95233°N, 74.34007°E	3m	thermokarst pond	sand, mosses	2m x 10m	0.5m
15	12.08.2014	72.94750°N, 74.33728°E	9m	thermokarst pond	silt, mosses	1.5m x 2m	1.5m
16	13.08.2014	72.93787°N, 74.39213°E	1m	thermokarst pond	silt, mosses	5m x 20m	2m
17	13.08.2014	72.95078°N, 74.38572°E	0m	thermokarst pond	sand, silt	10m x 30m	2m
18	14.08.2014	72.93840°N, 74.42920°E		thermokarst pond	silt, mosses	5m x 20m	1m
19	15.08.2014	72.93803°N, 74.34598°E	-9m	thermokarst pond	silty sand, mosses	20m x 40m	1m
20	15.08.2014	72.94862°N, 74.46823°E	0m	thermokarst pond	clay	20m x 45m	1.5m
21	15.08.2014	72.98183°N, 74.51590°E	7m	lake	sand	450m x 550m	1.5m

**Table 2.** Main characteristics of microcrustacean communities in the observed water bodies.

**Site No.**	**Dominant species** (% of total abundance)	**Subdominants** (% of total abundance)	**Total number of species**
1	*Scapholeberis mucronata* (20%) + *Chydorus sphaericus* (19,2%)	*Simocephalus vetulus* (15,8%)	10
2	*Leptodiaptomus angustilobius* (83,3%)	*Polyphemus pediculus* (12,5%)	3
3	*Polyphemus pediculus* (90,8%)	*Chydorus sphaericus* (6,5%)	5
4	*Leptodiaptomus angustilobius* (99,7%)	-	4
5	*Leptodiaptomus angustilobius* (97,9%)	-	5
6	*Leptodiaptomus angustilobius* (70,4%)	*Chydorus sphaericus* (23,5%)	6
7	*Polyphemus pediculus* (62,1%)	*Chydorus sphaericus* (15,3%)	7
8	*Polyphemus pediculus* (57,1%)	*Chydorus sphaericus* (28,6%) + *Leptodiaptomus angustilobius* (14,3%)	3
9	*Chydorus sphaericus* (51%)	*Polyphemus pediculus* (36%)	4
10	-	-	0
11	*Chydorus sphaericus* (100%)	-	1
12	*Leptodiaptomus angustilobius* (100%)	-	1
13	*Leptodiaptomus angustilobius* (86,2%)		4
14	*Polyphemus pediculus* (72,2%)		2
15	-	-	0
16	*Chydorus sphaericus* (95,7%)	*Alona affinis* (4,3%)	2
17	*Bosmina longirostris* (80%)	*Graptoleberis testudinaria* (20%)	2
18	*Leptodiaptomus angustilobius* (66,7%)	*Cyclops vicinus* (25%)	4
19	*Chydorus sphaericus* (93,8%)	-	4
20	*Leptodiaptomus angustilobius* (88,5%)	*Chydorus sphaericus* (8,8%)	3
21	*Polyphemus pediculus* (61,2%)	*Leptodiaptomus angustilobius* (24,3%)	6
